# Modulation of Diabetes Mellitus-Induced Male Rat Reproductive Dysfunction with Micro-Nanoencapsulated* Echinacea purpurea* Ethanol Extract

**DOI:** 10.1155/2018/4237354

**Published:** 2018-08-30

**Authors:** Chien-Feng Mao, Xiu-Ru Zhang, Athira Johnson, Jia-Ling He, Zwe-Ling Kong

**Affiliations:** Department of Food Science, National Taiwan Ocean University, 2 Pei-Ning Road, Keelung 20224, Taiwan

## Abstract

Diabetes mellitus is a major health problem that affects a patient's life quality throughout the world due to its worst complications. It was recognized that chronic hyperglycemia with oxidative stress was the major cause of male infertility.* Echinacea purpurea* ethanol extract (EE) contains phenolic acid and isobutylamides had been proven to ameliorate diabetic complications. Chitosan/silica nanoparticles are well-known in the medicinal field because of its controlled release and drug delivery properties. This study was aimed at investigating whether the EE encapsulated chitosan/silica nanoparticle (nano-EE) can enhance the amelioration of male infertility. Our results indicated that the average size of nano-EE was 218 ± 42 nm with an encapsulation efficiency of 66.9% and loading capacity of 39.9%. The reduction in oxidative stress and antioxidant activity of nano-EE was observed in LC-540 cells. In* in vivo* experiment, 33 mg/kg of streptozotocin (STZ) was used to induce diabetes in male Sprague-Dawley rats. Diabetic rats were treated with nano (465 mg/kg), nano-EE 1 (93mg/kg), nano-EE3 (279mg/kg), nano-EE5 (465 mg/kg), and metformin (Met) (200 mg/kg) for 7 weeks. The results show that the nano-EE5 can improve hyperglycemia, insulin resistance, and plasma fibroblast growth factor 21 (FGF 21) resistance. It was also confirmed that nano-EE5 significantly improved the testis tissue structure, increasing sperm quality and DNA integrity as well as reducing reactive oxygen species level.

## 1. Introduction

Type 2 diabetes mellitus is a complex disease characterized by the improper use of insulin by the pancreatic beta cells associated with hyperglycemia and insulin resistance [1]. Oxidative stress is responsible for the onset of several complications of diabetes such as vascular diseases, kidney damage, and reproductive dysfunction [2]. Both diabetic patients and animals commonly experience reproductive system deficiencies like testicular dysfunctions. Diabetes could also induce structural changes in the testis and spermatozoa and promote germ cell apoptosis, impairment of sperm parameters, and hormonal changes that finally results in infertility [3].

*Echinacea purpurea *is also known as purple coneflower originated in North America and was brought to Europe in the late 19th century. The most active compounds of* E. purpurea* are isobutylamides and polyphenols-caffeic acid derivatives such as caftaric acid, chlorogenic acid, cynarin, echinacoside, and cichoric acid [4]. Extracts and dietary supplements from this plant exhibited anti-immunosuppressant [5], antioxidative [6], anti-inflammatory [7], antibacterial [8], antiviral [9], and anticancer [10] properties. Despite all these wide spectra of pharmacological properties, the use of* E. purpurea* extract in the biomedical application field is limited due to its bitter taste and astringent and low aqueous solubility as well as low oral bioavailability [11]. Polyphenols are known as the most active compounds of* E. purpurea*. They are the secondary metabolites possessing radical scavenging activity towards reactive oxygen species (ROS). Because of the fast release, destruction against environmental stress, low bioavailability, low permeation, and low solubility, the direct use of phenolic compounds was limited [12].

One of the promising ways to circumvent these problems is through nanoencapsulation by using nanocarriers. It acts as a controlled drug delivery system and can improve the oral bioavailability of the lipophilic and hydrophobic drugs. There are several methods adopted for nanoencapsulating phenolic compounds into a carrier system. The methods are primarily categorized into physical methods (spray-drying, fluid bed coating, centrifugal extrusion, etc.), physiochemical methods (spray-cooling, ionic gelation, solvent evaporation extraction, etc.), and chemical methods (*in situ* polymerization, interfacial polymerization, etc.) [13]. Several investigations have been carried out using polymers (natural and synthetic) for the development of nanoparticles to improve the oral bioavailability [14]. Nowadays, the studies based on nanotechnology are expanded in almost all research fields. Nanoparticles got a lot of attention in drug delivery research due to its versatility in targeting tissues and enabling deep molecular targets [15].

Chitosan is an N-acetylated derivative of chitin widely used as a building material for nanoparticles [16]. It has excellent biodegradable, biocompatible, nontoxic, nonimmunogenic properties and it prolongs drug release time in the gastrointestinal tract [17]. Most importantly, chitosan is digested by chitosanase enzymes secreted by microorganisms of the intestine after oral ingestion. Silica is another material usually used for the building up of nanoparticles and is obtained from diatom skeletons, sponge speckles, and animal dietary silicon [16]. The potential applications of silica in nanobiotechnology include the encapsulation of biomolecules, drug delivery, and gene transfer [18].

This study has investigated the preparation and detailed characterization of chitosan/silica encapsulated EE and also performed the* in vitro *study to find out the effect of nano-EE on H_2_O_2_-induced oxidative stress in LC-540 and further performed the* in vivo* study to investigate the effect of the above said particle on diabetes-induced male rat reproductive dysfunction.

## 2. Materials and Methods

### 2.1. Reagents

Chitosan was purchased from Lytone Enterprise, Inc., Taipei, Taiwan [degree of deacetylation (DD) = 81%, molecular weight (Mw) = 200 kDa].* Echinacea purpurea* was purchased from Taiwan Direct Biotechnology Corp., (Hsinchu, Taiwan). Sodium acetate and acetic acid were purchased from Zhenfang Company (Taipei, Taiwan). Sodium silicate was purchased from Wako Chemical (Osaka, Japan). Nitroblue tetrazolium (NBT), 3-(4,5-dimethylthiazol-2-yl)-2,5-diphenyltetrazolium bromide (MTT), sodium nitrite, streptozotocin (STZ), acridine orange (AO), and DCFH-DA were purchased from Sigma (St. Louis, MO, USA). Metformin HCl was purchased from TCM Biotech International Corp. (Taipei, Taiwan). Alanine transaminase (ALT), aspartate transaminase (AST), blood urea nitrogen (BUN), creatinine, and glucose ELISA kits were purchased from Randox (Colorato, USA). Interleukin-1 beta (IL-1*β*), inflammatory cytokines tumor necrosis factor-alpha (TNF-*α*), FGF 21, and Testosterone ELISA kits were purchased from Abcam (Connecticut, US). Insulin ELISA kit was purchased from Mercodia (Uppsala, Sweden). Plasma AGE with AGEs determine ELISA kit. Dulbecco's modified eagle's medium, nutrient mixture F12 (DMEM-F12), and Trypsin, RPMI 1640, were purchased from Gibco (Carlsbad, California, USA). Fetal bovine serum (FBS) was purchased from PAA (Pasching, Austria). Dulbecco's phosphate-buffered saline was purchased from Nissui Pharmaceutical Co., Ltd. (Tokyo, Japan). LC-540 (rat testicular Leydig cell lines) came from the Food Industry Research and Development Institute (Hsinchu, Taiwan).

### 2.2. Preparation of* Echinacea purpurea* Extract (EE)

Experiment sample of* Echinacea purpurea* was provided by Taiwan Direct Biotechnology Corp.* Echinacea purpurea *were extracted with 70% ethanol and later freeze-dried (Freeze drying system FD 4.5 12XL, Kingmech Co., Ltd., Taipei, Taiwan). The acquired EE powder yield approximately 10%.

#### 2.2.1. High-Performance Liquid Chromatography (HPLC) Analysis for the Determination of Alkylamides and Total Phenolic Acid in EE.

Series III LC pump (Scientific Software, Inc., Pleasanton, CA, USA) was used as a system for performing HPLC analysis. 10 *μ*l of EE was injected onto a Phenomenex C18 column (250 mm × 4.6 mm, 5 *μ*m) (Torrance, CA, USA) at 35°C. Mobile phase A was acetonitrile: water: phosphoric acid (100:900:1) and mobile phase B was acetonitrile: water: phosphoric acid (900:100:1). A gradient solvent delivery was used with A maintained at 100% (30°C) for 5 minutes. The proportion of B decreased to 0% and the column was washed with 100% A for 10 min until the next injection. The flow rate was 1.2 mL/min, and the wavelength of the UV-visible detector was set (330 nm for total phenolic acids and 254 nm for alkalamides). The data were collected (HyperQuan Inc., VUV-20, Colorado Springs, Colorado, USA) and detected (Showa Denko, Japan).

### 2.3. EE Loaded Chitosan/Silica Nanoparticle Preparation

According to the previously reported method [19], chitosan/silica nanoparticles were prepared by mixing silicate solution and a chitosan solution. The silicate solution was prepared by dissolving sodium silicate in 0.05 M sodium acetate solution (0.7% w/w). Chitosan solution was prepared by dissolving chitosan in 0.1 M acetic acid solution (0.7% w/w). Chitosan and silica mixing ratio were 10:1 and the acidity of both solutions was adjusted to pH 5.6. EE was added to the chitosan/silica solution. The ratio of solution and EE was 11:1 and stirred homogeneously at room temperature for 30 minutes. After stirring, the mixture was centrifuged (high speed centrifuge, Hettich CR-12, Germany) at 6000 ×g for 30 minutes and the supernatant was freeze-dried (freeze drying system FD 4.5 12XL, Kingmech Co., Ltd., Taipei, Taiwan). The acquired freeze-dried product was nano-EE [20].

### 2.4. Particle Size, Zeta Potential, and Morphology Analysis

The shape and size of the particle were determined by scanning electron microscope (SEM) imaging [19]. The conductive double-sided tape was attached on the surface of a test plate. The sample was dissolved in 70% of alcohol for 30 minutes. Later, the sample was dropped on the tape and was dried in the convection oven for 24 hours. The sample was sputter-coated with a thin layer of gold and observed under SEM (Olympus Hitachi, Tokyo, Japan). Zeta potential and particle size (for confirmation) were determined by using Dynamic Light Scattering (DLS) (Zetasizer Nano ZS, Malvern Instruments, United Kingdom) method [21]. The sample was diluted to a thousand times with deionized water and was shaken for 1 minute in an ultrasonic water bath for getting homogenous dispersion. 700 *μ*L of sample was injected into the disposable capillary sample cell (DTS1061) for the analysis.

### 2.5. Preparation of Encapsulation Efficiency and Loading Capacity

The encapsulation efficiency and loading capacity were determined by HPLC (Series II LC pump, SSi, USA, HyperQuan Inc. VUV-20, Shodex RI-71 detector, Showa Denko, Japan) analysis. The analysis was conducted by detecting the content of caftaric acid of nano-EE and EE. The encapsulation efficiency and loading capacity of nano-EE were calculated by the following formulas [20]:

(1) $$\begin{array}{c} \text{Encapsulation efficiency}=\left[\;\frac{\left(A-B\right)}{A}\times 100\%\;\right] \end{array}$$

A is total amount of caftaric acid and B is free caftaric acid in the supernatant.

(2) $$\begin{array}{c} \text{Loading capacity}=\left[\;\frac{\left(A-B\right)}{C}\times 100\%\;\right] \end{array}$$

A is total amount of caftaric acid, B is free caftaric acid in the supernatant, and C is weight of the nanoparticles.

### 2.6. In Vitro Cell Experiments of Nano-EE and EE

#### 2.6.1. Cell Culture

The LC-540 were cultured in Dulbecco's modified eagle medium: nutrient mixture F-12 (DMEM-F12) medium supplemented with fetal bovine serum (FBS) and maintained at 37°C in a humidified incubator containing 5% CO_2_ [22].

#### 2.6.2. Cell Viability Assay

The cell viability assay was performed by using the 3-(4, 5-dimethylthiazol-2-yl)-2, 5-diphenyltetrazolium bromide (MTT) reagent. The samples were dissolved in dimethyl sulfoxide (DMSO) and diluted in cell culture medium. The final concentration of cells in DMSO was 0.1%. The different doses were determined by weighing method. In control experiments, this concentration did not have any effects on the measured parameters. The cells were cultured in 96 well plate at a concentration of 4 × 10^5^ cells/well and stimulated with hydrogen peroxide (H_2_O_2_). After 24 hours of preconditioning, the culture medium was aspirated and cells were treated with a variety of concentrations of the sample for 24 hours. Subsequently, 100 *μ*l of MTT dye (1 mg/ml) was added to the culture and further incubated for 4 hours at 37°C. The cell viability was calculated by measuring the absorbance at 540 nm (ELISA Reader, Thermo Fisher 1510, Germany) [23].

#### 2.6.3. Effect of EE on Nitric Oxide (NO) Production in H_2_O_2_-Induced LC-540 Cells Oxidative Stress Model

Nitrite as the end product of NO assay was measured by using the Griess reagent. Briefly, culture supernatants (50 *μ*l) were mixed with 50 *μ*l of the Griess reagent and the nitrite concentration in the culture supernatant was obtained by measuring the absorbance at 570 nm. The nitrite concentration was determined with reference to the standard curve using sodium nitrite [24].

### 2.7. Animal Model Design

5-week-old Sprague-Dawley (SD) rats has purchased form BioLASCo Taiwan. Each rat was housed individually in cages under controlled temperature (23 ± 1°C) and humidity (50 ± 10%) with lighting on a 12 h light/12 h dark cycle. Feed and water were provided as ad libitum. Each rat was domesticated in the first week and the laboratory Rodent Diet 5001 was given as the main diet. After domestication, rats were divided into two groups such as control group (Con) that were fed with Lab Diet 5001 and diabetic group that were fed with high-fat diet (HFD) (40% fat calories) for the entire experiment. After feeding with HFD for 4 weeks, the diabetic group rats were injected with a low dose of streptozotocin (STZ) to induce diabetes mellitus (DM). After 1 week, oral glucose tolerance test (OGTT) was conducted to determine the successful induction of DM. Later, DM group were divided into 6 groups DM group without treatment, Met group (DM treated with metformin), nano group (DM with chitosan/silica nanoparticle), and three experimental nano-EE groups (DM with low, medium and high dose of nano-EE; 93, 279 and 465 mg/kg B.W, respectively). Treatments were given until the end of the experiment. During the experiment, the body weight and food and water intake were monitored and recorded between every three days. All experimental rats were sacrificed at the age of 18 weeks. Blood samples were collected in heparinized tubes by hepatic portal vein sampling. Blood plasma was separated by centrifugation at 3000 rpm at 4°C for 15 min. Body organs were weighed and frozen for further analysis.

#### 2.7.1. Postmortem Analyses/Analyses after the Sacrifice

(*1) Sperm Analysis.* The swim-up method was used to collect sperm from the epididymis. Epididymis was cut into 3 pieces, immersed in Roswell Park Memorial Institute (RPMI) medium and shaken in an orbital shaker at 100 rpm for 30 min. Epididymis with RPMI medium was centrifuged at 190 ×g for 5 min and incubated at 37°C in 5% CO_2_ incubator for 30 minutes. Finally, the sperms were collected from the supernatant and observed under the microscope for determining the sperm count, motility, and abnormality. Reactive oxygen species (ROS) damage was determined by using fluorescent dyes dichloro-dihydro-fluorescein diacetate (DCFH-DA) and flow cytometry [25]. The DNA fragmentation of sperm was noticed by using acridine orange (AO) staining method under the fluorescent microscope [26]. Nitric oxide (NO) and nitroblue tetrazolium (NBT) assay were conducted by using chemical assay methods [24, 27].

(*2) Testis Histology Analysis.* After sacrifice, the testicles were collected and were immersed in 10% formaldehyde. The testis was cut into 5 mm from the middle part with a scalpel. The group was marked with pencil on the lid and soaked back into formalin. Specimens were later sent to Rapid Science Co., Ltd. at room temperature for paraffin-embedding and hematoxylin & eosin (H&E) staining.

*(3) Preparation of Testis Homogenates.* 100 mg of testicular tissue was cut, rinsed with PBS, centrifuged for 1 min, and homogenized (Tissue homogenizer, Hitachi SCR 20BA, Tokyo, Japan) in 200 *μ*L of homogenizing solution. 800 *μ*L of PBS was added and the mixtures were chilled to −20°C for 24 hours, followed by −4°C thawing, frozen again to −20°C, then thawed, and centrifuged at 4°C (15000 ×g rpm) for 15 minutes [28]. Supernatants were collected and were stored at −80°C (−80°C Freezer, Nuaire, United Kingdom). The total protein content of homogenates was determined by Bradford assay.

*(4) Analysis of Testis Homogenates.* Inflammation levels in testis were determined by measuring tumor necrosis factor-*α* (TNF-*α*) and IL-1*β* content in homogenates [29].

### 2.8. Statistical Analysis

The data were analyzed using SPSS 22.0 software. All data were expressed as a mean ± standard error of measurement (SEM) (N= 8). Comparison among groups was made by one-way analysis of variance (ANOVA). Multiple comparisons of different groups were analyzed by Duncan's test at the value of* p* < 0.05 as significant level.

## 3. Results and Discussion

### 3.1. The Chemical Composition of Phytopharmacological Preparations and Encapsulation Efficiency of Nano-EE

**Figure 1:**
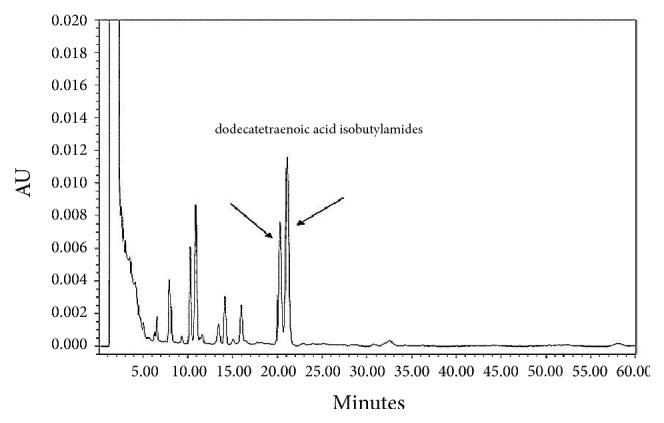
*The HPLC chromatograms of the alkylamides in EE.* The chemical profile of EE was detected at UV 254 nm.

**Figure 2:**
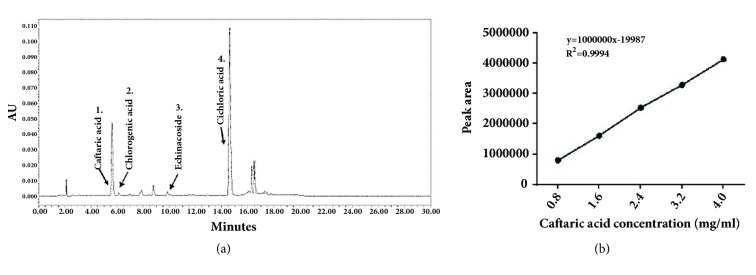
(a) The HPLC chromatograms of the total phenolic acids in EE (the chemical profile of EE was detected at UV 330 nm); (b) standard curve of caftaric acid.

*Echinacea purpurea* contained many active compounds such as polysaccharides, lipid compounds, isobutylamides, total polyphenols, flavonoids, and inorganic salts. The content of total polyphenols and alkylamides in EE depended upon the extracted parts, species, and the methods of extraction. Alkylamides are commonly found in the roots of Echinacea and are related to the effects of immunomodulation, anti-inflammation, and antidepression. The polyphenols are largely obtained from the floral part (also seen in stem and leaves) of Echinacea and are well-known to produce antioxidant activity. Previous studies show that the isobutylamides and total polyphenols compounds of EE had many pharmacological activities [4]. Increased resistance to oxidation stress was related to the number of hydroxyl groups on the phenol. The number of hydroxyl groups and scavenging activities is directly proportional [30]. The efficacy order of EE components was in the following manner: - echinacoside > cichoric acid > cynarin > chlorogenic acid > caffeic acid > caftaric acid [31]. The content of isobutylamides and total polyphenols of nano-EE and EE were evaluated by HPLC analysis. Dodecatetraenoic acid isobutylamide was identified from HPLC analysis (Figure 1). The total phenolic compounds which were detected from the HPLC analysis are caftaric acid, chlorogenic acid, echinacoside, and cichloric acid (Figure 2(a)) and the standard curve of caftaric acid was given in Figure 2(b). The isobutylamides of freeze-dried nano-EE and EE were 0.21 mg/g and 0.88 mg/g, respectively, while the total polyphenols of freeze-dried nano-EE and EE were 35.3 mg/g and 20.8 mg/g respectively (Table 1). In addition to this, the extraction yield of EE obtained was 7%. The loading capacity and encapsulation efficiency of nano-EE were about 39.9% and 66.9%, respectively. So the results confirm that nano-EE has good loading capacity and encapsulation efficiency (Table 1).

### 3.2. Characterization of Nano-EE

**Figure 3:**
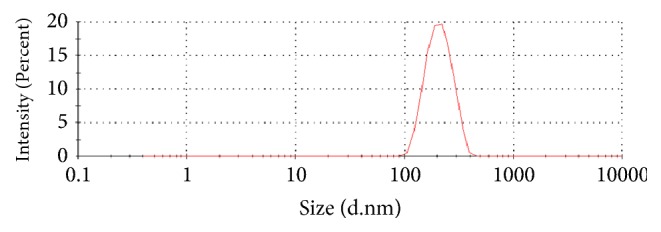
Dynamic light scattering (DLS) of nano-EE particle size.

Chitosan/silica nanoparticle has a small particle size, controlled release, and good stability and it can be used as potential drug delivery systems for biomedical applications [19]. The size and shape of the particle were determined by SEM analysis. The average particle size (confirmation), dispersion, and zeta potential of the nano-EE were measured by DLS method. The average particle size of nano-EE obtained from DLS method was 278 ± 21 nm (Table 2) (Figure 3). From the size distribution data, it was evident that a minor portion of the nanoparticle population has higher particles size.

The nanoparticles showed high polydispersity index (PDI) value in the range of 0.37±0.01 (Table 2). PDI is a kind of particle size dispersion index indicating the homogeneity of the carrier system [32]. The better PDI value was obtained for nano-EE group.

Zeta potential is an important parameter for interpreting the electrostatic potential near the surface of nanoparticles and predicting their degree of stability. It is a measure of the magnitude of charges on nanoparticles and also denoting the stability of the particles. The obtained zeta potential of the nanoparticles was −21.3 ± 0.55 mV (Table 2). The negative charge of zeta potential indicated that the nano-EE has a negative charge and is stable in water. These charged particles can interact more favorably with the cell membrane and thereby enhance the uptake of the particle.

**Figure 4:**
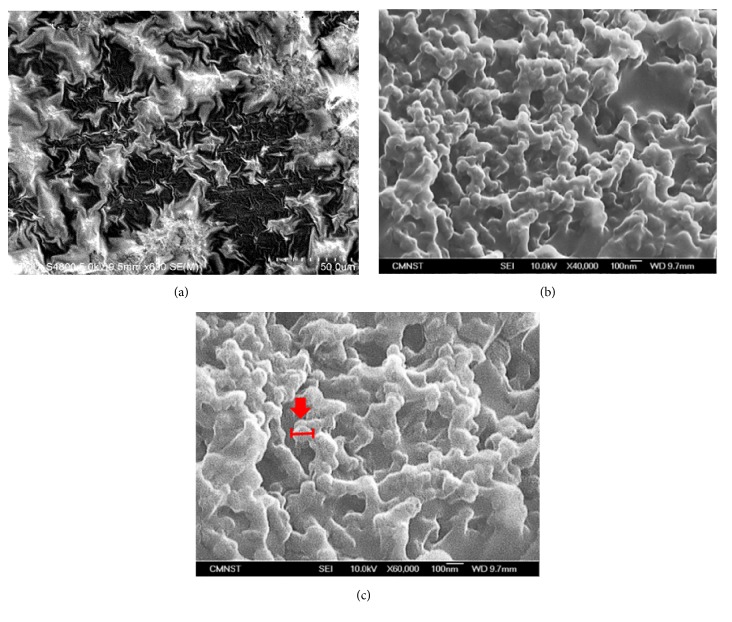
Scanning electron micrographs (SEM) of EE and nano-EE structures EE at 600 times magnification (a), nano-EE at 40,000 times magnification (b), and 60,000 times magnification (c).

Furthermore, with SEM analysis, the size and shape of the prepared nano-EE (Figure 4) were analyzed. The SEM investigation of the nano-EE showed that the average particle size of the nano-EE was 218 ± 42 nm in diameter. Particles with a size between 100 and 1000 nm belong to the micro-nanograde particles. However, From SEM (40000x) analysis, it was found that the particles were clustered in nano-EE group. At 60,000 times magnification, the results indicated that nanoparticles were appeared similar to small beads. Finally, it was founded that the particle size of the DLS was greater than that of the SEM. The reduction in the size of the particle was due to particle shrinkage during SEM analysis. It could be assumed that the size of the nanoparticle was reduced due to the loss of moisture content from the samples and the nanoparticles to get closer to each other. It was understood that the chitosan-silica nanoparticle was formed with poor dispersion and has spherical clusters with agglomeration.

### 3.3. Cell Experiments

#### 3.3.1. H_2_O_2_-Induced LC540 Oxidative Stress Model

**Figure 5:**
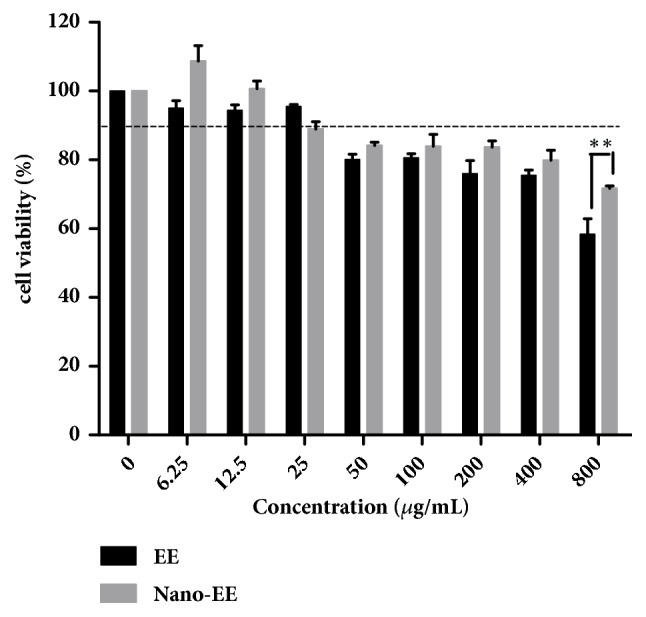
*Cell viability of LC-540 was treated with nano-EE and EE for 24 hours.* LC-540 cells were adjusted to 4 × 10^5^ cells/ml and treated with nano-EE and EE for 24 hours. The cells viability was analyzed by MTT assay. Results were shown by mean ± SEM (n = 3).* P* < 0.01 (*∗∗*) indicates significant differences between EE and nano-EE.

(*1) Protective Effect of Nano-EE on H*_2_*O*_2_*-Induced Cytotoxicity.* The cytotoxicity of nano-EE and EE in LC-540 cells was evaluated by MTT assay (Figure 5). The results show that there is not any significant difference in the cytotoxicity of nano-EE and EE groups at concentrations ranging from 0 *μ*g/mL to 25 *μ*g/mL. Thus, the highest concentrations of nano-EE available for LC-540 cell line were 25 *μ*g/mL. Cell viability of LC-540 cells with 800 *μ*g/mL dose of nano-EE and EE has a significant difference. The result indicated that nano-EE has a slow and sustained release of the EE solution.

**Figure 6:**
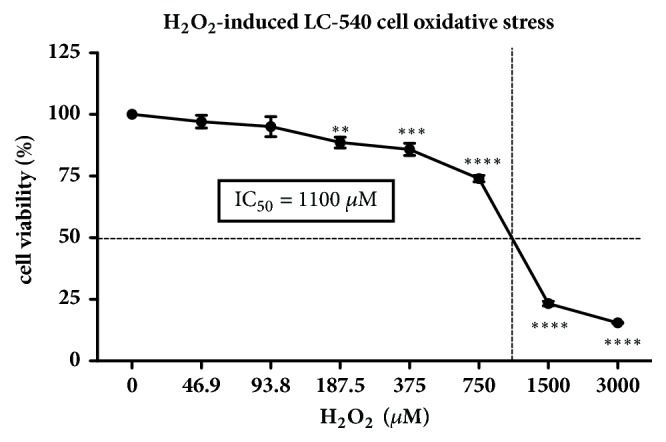
*Cell viability of various H*_2_ *O*_2_ *-induced LC-540 cell stress for 24 hours.* Cells were adjusted to 4 × 10^5^ cells/ml and treated with H_2_O_2_ for 24 hours. The cells viability was analyzed by MTT assay. Results were shown by mean ± SEM (n = 3). P < 0.0001 (*∗∗∗∗*), P < 0.001 (*∗∗∗*), and P < 0.01 (*∗∗*) versus 0 ng/ml H_2_O_2_.

**Figure 7:**
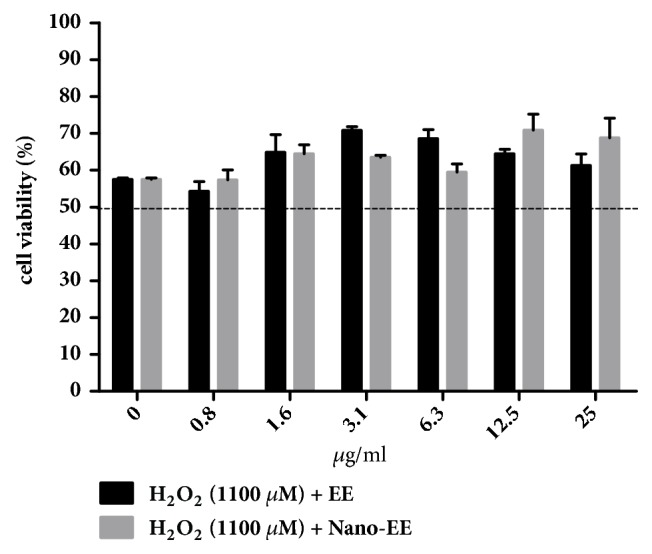
*LC-540 was treated with different EE, nano-EE, and H*_2_ *O*_2_ *for 24 hours.* Cells were adjusted to 4 × 10^5^ cells/ml were treated for 24 hours with EE, nano-EE, and H_2_O_2_ (1100 *μ*M). The cells viability was analyzed by MTT assay. It can decide the amount of the damage of the H_2_O_2_. Results were shown by mean ± SEM (n = 3).

**Figure 8:**
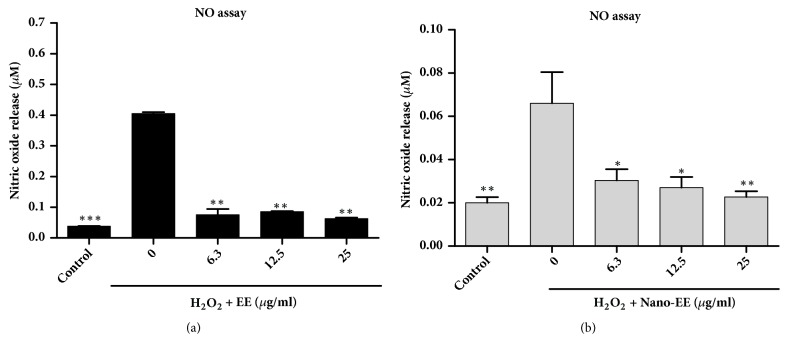
*Effects of EE and nano-EE on H*_2_ *O*_2_ *induced nitric oxide release for 24 hours by NO assay in LC-540.* Cells were adjusted to 4 × 10^5^ cells/ml were treated for 24 hours with EE (a), nano-EE (b), and H_2_O_2_ (1100 *μ*M). The cells viability was analyzed by NO assays. It can decide the amount of the damage of the H_2_O_2_. Results were shown by mean ± SEM (n = 3).* P* < 0.01 (*∗∗*);* P* < 0.05 (*∗*) versus 0 ng/ml H_2_O_2_ + EE or nano-EE.

The cytoprotective effect of nano-EE (0, 0.8, 1.6, 3.1, 6.3, 12.5, and 25 *μ*g/mL) in H_2_O_2_-induced LC-540 cells was examined in Figure 6. Cell viability of LC-540 cells exposed to 1100 *μ*M H_2_O_2_ decreased below 60% and increased significantly to 70% in the nano-EE-treated group at 25 *μ*g/mL (Figure 7).

(*2) Effect of Nano-EE on H*_2_*O*_2_*-Induced NO Production.* Oxidative stress plays an important role in male infertility. The imbalance between ROS production and antioxidants activity might lead to a rise in ROS levels in the semen. High level of ROS affects the unique ability of the male germ cells to move forward and also upsets their ability to fertilize to the oocyte. The fertilizing ability of the human spermatozoa is inversely proportional to the sperm ROS production. H_2_O_2_ is one of the major ROS associated with the oxidative stress. It readily penetrates into cells and reacts with intracellular metal ions such as iron or copper to generate highly reactive O_2_- and nitric oxide (NO-) radicals [33]. It was identified that a strong inhibition of cell growth by H_2_O_2_ and the protective effect of nano-EE against H_2_O_2_-induced oxidative stress in LC-540 cells. It was further observed that nano-EE and EE could protect the LC-540 from H_2_O_2_-induced oxidative stress and reduced NO production. The results indicated that nano-EE was capable of reducing H_2_O_2_-induced cytotoxicity. The NO release of nano-EE and EE (0, 6.3, 12.5, and 25 *μ*g/mL) in H_2_O_2_-treated LC-540 cells was evaluated after 24 hours (Figure 8). The results show that the production of NO was significantly reduced with an increase in the concentration of nano-EE and EE. As compared to nano-EE, EE has a significant difference in inhibiting NO production. The highest concentration (25 *μ*g/mL) of nano-EE and EE exhibited more NO reduction than other concentrations. Therefore, nano-EE and EE can significantly reduce NO production and thereby lower the oxidative stress. 

### 3.4. Animal Experiments

#### 3.4.1. Evaluation of the Body Weight, Food Intake, and Water Intake in Diabetic Rats Fed with Nano-EE

The STZ-induced experimental diabetic animal model was characterized by hyperglycemia, increased food, and water intake, and decreased body weight has been chosen for the present study [34]. From this study, it could be understood that the weight loss has happened when fed with nano-EE3 and nano-EE5 may be caused due to an increase in the metabolism of the body.

Metformin (Met) which is an important antihyperglycaemic agent commonly used for the treatment of type 2 diabetes mellitus has the ability to decrease the hepatic glucose production via a mild and transient inhibition of the mitochondrial respiratory chain complex I. In addition, it results in the reduction of hepatic energy status and activates AMP-activated protein kinase (AMPK) a cellular metabolic sensor, providing a generally accepted mechanism for the action of metformin on hepatic gluconeogenesis [35].

**Figure 9:**
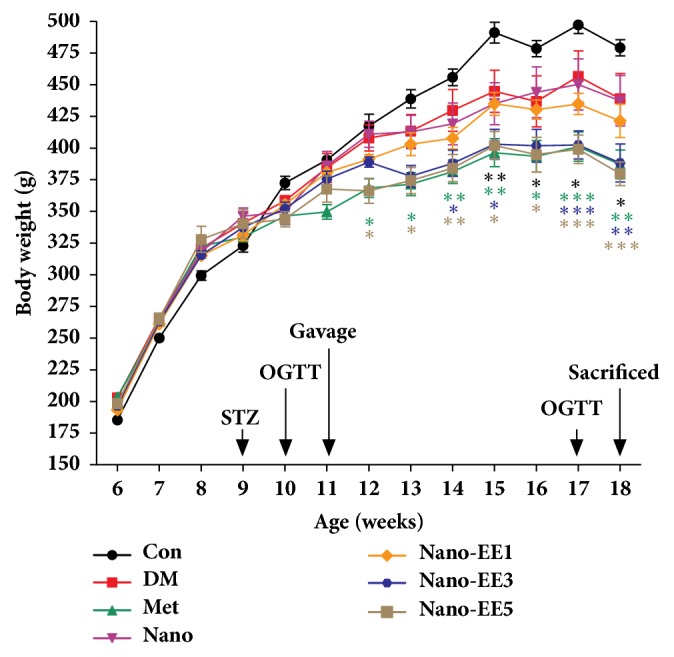
*Body weight of rats during experiment.* Data are shown as the mean ± SEM (n = 5 rats/group). Con: control; DM: diabetes; Met: diabetes + 200 mg/kg per day metformin; Nano: diabetes + 465 mg/kg per day chitosan and silica; Nano-EE1: diabetes + 93 mg/kg per day EE; Nano-EE3: diabetes + 279 mg/kg per day EE; Nano-EE5: diabetes + 465 mg/kg per day EE. P < 0.001 (*∗∗∗*), p < 0.01 (*∗∗*), and p < 0.05 (*∗*) versus DM.

We recorded the body weight of each rat from 6 weeks old to 18 weeks old (Figure 9). Rats were induced to diabetes at the age of 9 weeks by using STZ. The results show that the body weight of control (Con) group was increased in every week while the body weight of diabetic-induced groups was significantly lower than that of the control group. The metformin (Met) and different dose of nano-EE were administered at the age of 18 weeks. The result showed that there was a significant reduction in the body weight of Met, nano-EE3, and nano-EE5 in comparison with DM group and nano groups, though no significant differences were observed within these three groups.

**Figure 10:**
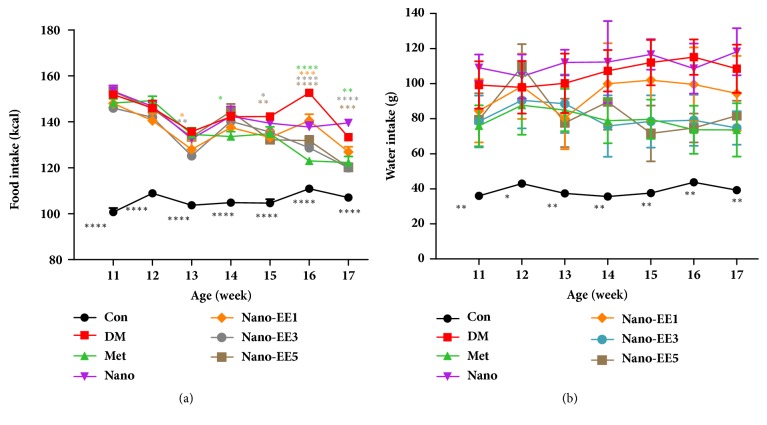
*Effects of nano-EE on food intake (a) and water intake (b) during 6 weeks in diabetic rats.* Data were shown as the mean ± SEM (n = 5 rats/group). Con: control; DM: diabetes; Met: diabetes + 200 mg/kg per day metformin; Nano: diabetes + 465 mg/kg per day chitosan and silica; Nano-EE1: diabetes + 93 mg/kg per day EE; Nano-EE3: diabetes + 279 mg/kg per day EE; Nano-EE5: diabetes + 465 mg/kg per day EE. P < 0.0001 (*∗∗∗∗*), P < 0.001 (*∗∗∗*), p < 0.01 (*∗∗*), and p < 0.05 (*∗*) versus DM.

The food and water intake of Con was lower than that of diabetes-induced groups (Figure 10). At the age of 11 weeks, the calories and water intake of DM group were relatively higher than the Con group. At the age of 16 weeks, there were not any significant differences observed in the calories and water intake of DM, nano, and nano-EE1 groups. The Met, nano-EE3, and nano-EE5 groups show relatively lower intake of calories and water. In the final week, lowered intake of calories and water were observed in each group by OGTT.

#### 3.4.2. Effect of Plasma Biochemical Parameters in Diabetic Rats

**Figure 11:**
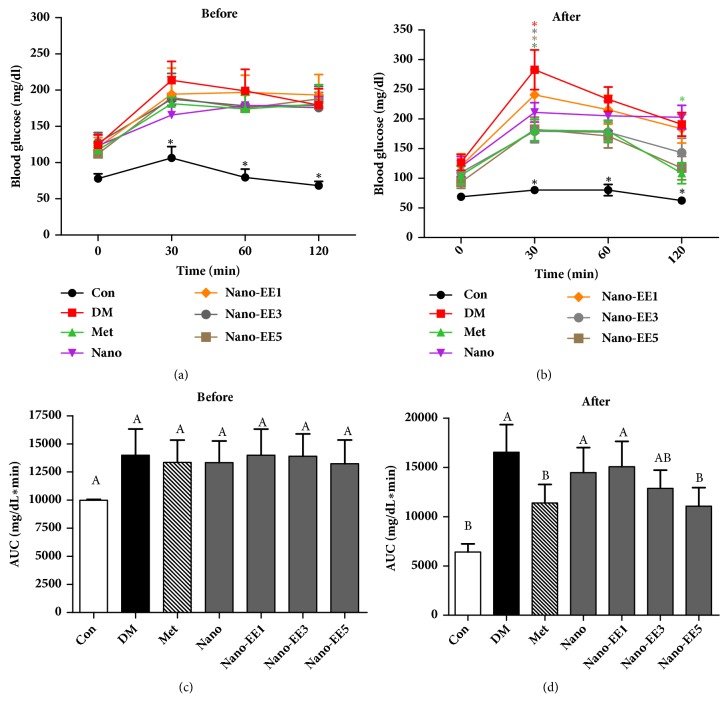
*Effects of nano-EE on oral glucose tolerance test (OGTT) (after oral glucose load of 2 g/kg) (a-b) and the area under curve (AUC) (c-d) in diabetic rats and before and after 7 weeks.* Data were shown as the mean ± SEM (n = 5 rats/group). Con: control; DM: diabetes; Met: diabetes + 200 mg/kg per day metformin; Nano: diabetes + 465 mg/kg per day chitosan and silica; Nano-EE1: diabetes + 93 mg/kg per day EE; Nano-EE3: diabetes + 279 mg/kg per day EE; Nano-EE5: diabetes + 465 mg/kg per day EE.* p* < 0.05 (*∗*) versus DM. The values with different letters (A-B) are significantly different (*p* < 0.05) as analyzed by Duncan's multiple range test.

(*1) Glucose Tolerance Test (OGTT) and the Area under the Curve (AUC) in Diabetic Rats after 7 Weeks.* OGTT is known as a marker for diagnosing diabetes. It was usually carried out after overnight fasting or glucose loading. The plasma glucose level was determined after 7 days of STZ administration (Figure 11(a)). When compared to the Con group, the fasting glucose level of other groups was elevated to above 100 mg/dl. It was observed that, after 2 hours, the blood glucose level of STZ-induced groups was greater than 140 mg/dl. The blood glucose level of STZ-induced groups was not significantly lowered to normal values and the AUC of blood glucose was also increased (Figure 11(c)). So, the results indicated that rats were successfully induced with diabetics. The blood glucose level of DM and nano groups has no any significant reduction at 120 min (Figure 11(b)). But, in the case of Met and nano-EE5 treatments, the blood glucose levels were lowered when compared to the DM group (Figure 11(a)). Furthermore, we found that the AUC of blood glucose in the nano-EE5 group was lowered to the AUC of the Met group in a dose-dependent manner (Figure 11(d)). 

(*2) Effects of Plasma Insulin Level, Homeostatic Model Assessment-Insulin Resistance (HOMA-IR), Advanced Glycation End Products (AGEs), and Plasma Fibroblast Growth Factor 21 (FGF 21) in Diabetic Rats Fed with Different Doses of Nano-EE after 7 Weeks.* Insulin is a peptide hormone produced by the beta cells of the pancreas. It is functionalized to maintain the blood sugar level and protect the body from hyperglycemia (sugar level high) and hypoglycemia (low sugar level) [36]. The insulin resistance is defined by HOMA-IR. The pathogenesis of diabetes is mediated by AGEs [37]. Diabetes can be caused by the binding of plasma glucose to the protein in the blood. After a series of chemical reactions, AGEs were produced as irreducible substances and can affect the normal functions of the DNA and gene expression of the cells. Accumulation of AGEs will generate a large amount of ROS and eventually cause an increase in oxidative stress [38]. FGF 21 is an endocrine hormone predominantly seen in liver, pancreas, and adipose tissue and is relatively less seen in other organs such as testis. The FGF 21 are functionalized to increase the glucose uptake in the adipose tissue, augment lipolysis, enhance production of ketone bodies in the liver, and regulate energy balance and physical stress responsiveness in humans. Serum FGF 21 is also a superior biomarker to other adipokines in predicting incident diabetes [39]. The level of plasma FGF 21 is increased in insulin-resistant states and correlates with hepatic and whole-body (muscle) insulin resistance in type 2 diabetes [40]. Diabetes and its related reproductive impairments have been widely studied, but the exact mechanisms for the reproductive dysfunction in males are not completely understood.

After the administration of different doses of nano-EE for 7 weeks, the levels of plasma insulin, HOMA-IR, advanced glycation end products (AGEs), and plasma FGF 21 were determined (Table 3). The results showed that DM group possesses insulin over secretion. No significant difference was observed between nano and DM group. But, nano-EE5 exhibited a reduction in insulin secretion. The significant reduction of insulin secretion was observed in the Met group. Furthermore, DM group and nano-EE1 had significantly increased the level of HOMA-IR and AGEs compared to Con group. Treatment with nano-EE5 effectively improved the HOMA-IR and AGEs levels to as low as the Met group. Finally, the FGF 21 level in DM group significantly increased. Nano-EE and Met group can be reduced the FGF 21 level in a dose-dependent manner, but no significant difference among the groups was observed. 

*(3) Effects of Plasma ALT, AST, Urea, and Creatinine in Diabetic Rats Fed with Different Doses of Nano-EE after 7 Weeks.* Elevation in the concentration of aspartate aminotransferase (AST) and alanine aminotransferase (ALT) are seen in diabetic condition. The level of urea and creatinine is higher in diabetes condition and it is known as a significant marker for renal dysfunction [41]. After the administration of different doses of nano-EE for 7 weeks, the activities of plasma enzymes AST and ALT were significantly higher in the diabetic group when compared to the Con group (Table 4). The treatment with the different dose of nano-EE and Met significantly prevented the rise of AST and ALT activities under diabetic condition. Levels of urea and creatinine are also increased significantly in diabetic group when compared to Con group. However, the administration of nano-EE5 and Met prevented the increased urea and creatinine levels.

#### 3.4.3. Evaluation of Nano-EE Reproductive Function in Diabetic Rats

(*1) Effects of Parameters on Sperm Count, Motility, Abnormality, and DNA Integrity by Acridine Orange (AO) Staining in Diabetic Rats.* Increase in the incidence of diabetes mellitus in worldwide in men is associated with subfertility and infertility. Long-term diabetes mellitus with uncontrolled hyperglycemia is responsible for the testicular dysfunction and resulting in the reduction of fertility potential. Later, it was described that STZ-induced diabetes mellitus in the male rats had altered sex behavior and diminished reproductive organ weight, testicular sperm content, and epididymal sperm content, as well as sperm motility. Increased sperm DNA damage was also reported in diabetic men at reproductive age associated with high oxidative stress resulting from sperm over exposure to glucose [42]. The mechanisms of sperm nuclear DNA damage in diabetic men promoted by ROS are suggested to be mediated through the activity of AGEs. These AGEs accumulate in the reproductive tract of diabetic men. Moreover, increased oxidative stress and higher DNA fragmentation are interconnected with apoptosis [42].

After the administration of different doses of nano-EE for 7 weeks, parameters such as sperm count, motility, abnormality (Figure 13), and DNA integrity were determined (Figures 14 and 15). Results show that a significant reduction in sperm count and motility as well as an increased abnormality was observed in the DM group when compared to the Con. But, Met has significantly increased the sperm count and motility and reduced the sperm abnormality. Treatment with nano-EE5 improved the sperm motility and abnormality but there were not any improvements seen in nano group (without EE).

The appearance of the sperm was observed the under microscope (Inverted Phase Contrast Microscope, Olympus IX-71, Tokyo, Japan) (Figure 14). Results showed that significant abnormalities such as the coiled tail, detached head, and aggregated sperm were spotted in DM and nano groups. Treatment with the Met and nano-EE5 groups shows better results as compared to the DM group and nano group.

Finally, based on the properties of the fluorochrome acridine orange (AO), which fluoresces green when bound to native deoxyribonucleic acid (DNA) (double-stranded and normal) and red when bound to denature DNA (single-stranded). Orange and red stained spermatozoa (orange and red arrows) were the abnormal sperm (denatured DNA) while green stained (green arrows) spermatozoa were considered to be normal (nondenatured DNA). Results showed that when compared to the Con group, the significant sperm DNA damage was seen in DM and nano group. Treatment with the Met and nano-EE5 groups were shown better improvements (Figure 15). The quantitative analysis of the sperm DNA integrity in Con and various experimental animals by acridine orange (AO) staining was shown in Figure 16. The percentage of the apoptotic cell was decreased in nano-EE5 group compared to nano, Met, and DM groups.

(*2) Effects of Nano-EE on the Sperm Production of ROS, NO, and NBT after 7 Weeks of Treatment in Diabetic Rats. *ROS production is known as the major process for oxidative stress during diabetes condition. Generation of ROS was determined by using DCFH-DA as a probe (Figure 17(a)). ROS levels were significantly increased in DM group when compared to the Con group. But, treatment with nano-3EE and nano-5EE significantly reduced ROS level in the sperm. Nano group did not show any improvement in ROS level.

The production of NO and superoxide anion in sperm was observed in diabetic rats (Figures 17(b) and 17(c)). The production of NO and superoxide anion in the DM group was significantly increased when compared to the Con group. Treatment with Met and nano-EE5 could reduce their productions. The reduction of NO and superoxide anion in Met was most significant, followed by the nano-EE5 group. These results are suggesting the anti-inflammatory potential of Met and nano-EE5 groups.

*(3) Effects of Nano-EE and EE on Diameters Seminiferous Tubules (DST), Germinal Cell Layer Thickness (GCLT), the Area of Seminiferous Tubules (AST), and Area of the Seminiferous Lumen (ASL) after 7 Weeks of Treatment.* The interior of the testis is composed of many seminiferous tubules. These seminiferous tubules are supported by the Sertoli cell and the Leydig cell exists in the space between the seminiferous tubules. Sertoli cell and Leydig cell are responsible for the regulation of androgen concentration and spermatogenesis along with sperm maturation. The immature sperm cells are located in the basement membrane while the mature sperm is located in the centre of the lumen [42].

**Figure 12:**
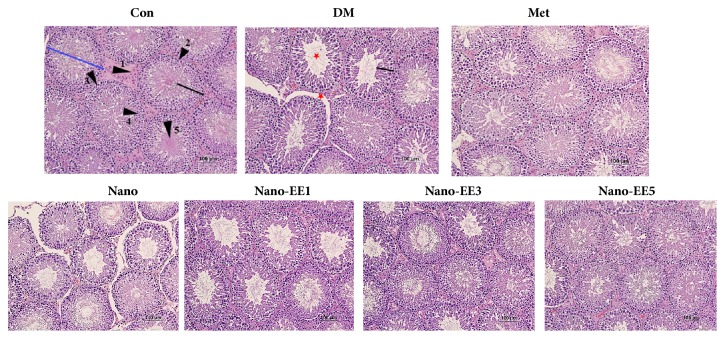
*Effects of nano-EE on seminiferous tubules of testis in diabetic rats after 7 weeks of treatment.* Representative images of hematoxylin and eosin (H&E) sections in the testis of each group (magnifications = 40X). Arrow 1: Leydig cells, arrow 2: Sertoli cell, arrow 3: spermatogonia, arrow 4: spermatocyte, arrow 5: sperm, blue line: diameters of seminiferous tubules (DST), black line: germinal cell layer thickness (GCLT), and star: tubular shrinkage and extensive intertubular area.

**Figure 13:**
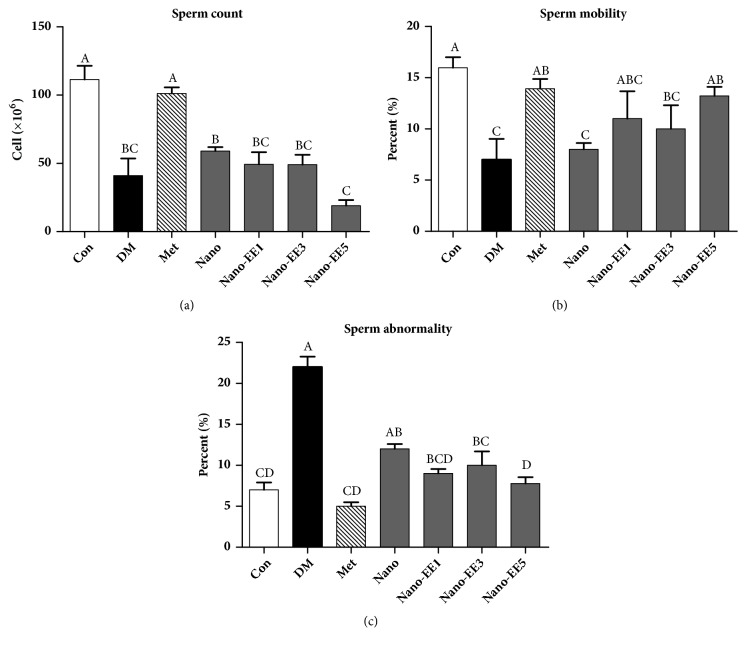
*Effects of sperm parameters, (a) count, (b) mobility, and (c) abnormality, in diabetic rats fed different doses of nano-EE in diabetic rats after 7 weeks.* Data were shown as the mean ± SEM (n = 5 rats/group). Con: control; DM: diabetes; Met: diabetes + 200 mg/kg per day metformin; Nano: diabetes + 465 mg/kg per day chitosan and silica; Nano-EE1: diabetes + 93 mg/kg per day EE; Nano-EE3: diabetes + 279 mg/kg per day EE; Nano-EE5: diabetes + 465 mg/kg per day EE. The values with different letters (A-D) are significantly different (p < 0.05) as analyzed by Duncan's multiple range test.

**Figure 14:**
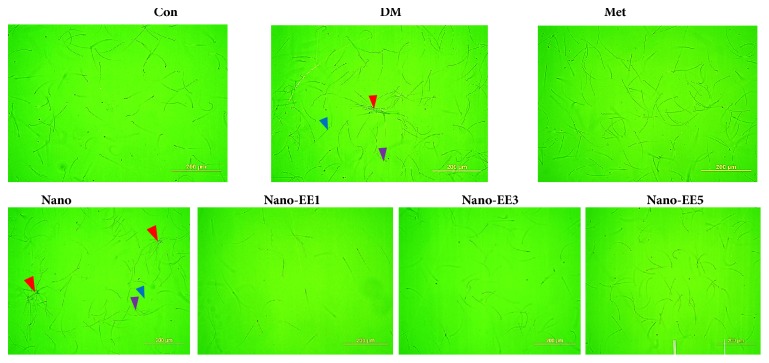
*Effects of nano-EE on sperm morphology seen in diabetic rats epididymal after fed different doses of nano-EE in diabetic rats after 7 weeks.* Different sperm abnormalities in different dose of nano-EE-treated diabetic rats. Purple arrow: coiled tail, blue arrow: detached head, and red arrow: aggregated sperm.

**Figure 15:**
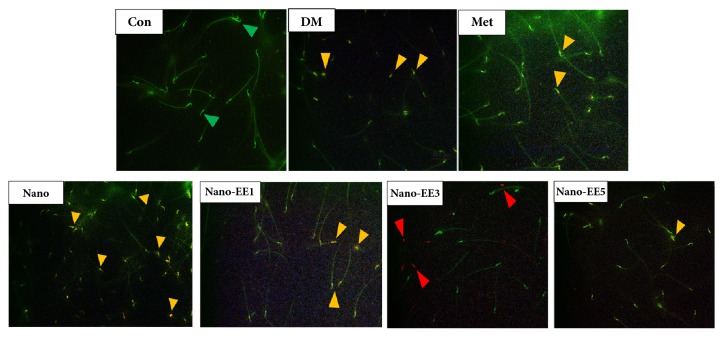
*Evaluation of the sperm DNA integrity in control and various experimental animals by acridine orange (AO) staining.* Orange and red stained spermatozoa (orange and red arrows) were abnormal sperm (denatured DNA) while green stained (green arrows) spermatozoa were considered to be normal (nondenatured DNA). For interpretation of the references to color in this figure legend, the reader is referred to the histogram of Photoshot.

**Figure 16:**
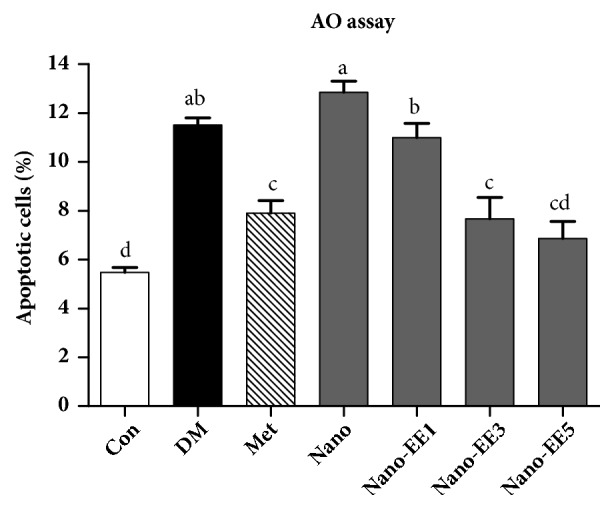
*Quantitative analysis of the sperm DNA integrity in control and various experimental animals by acridine orange (AO) staining.* For interpretation of the references to color in this figure legend, the reader is referred to the histogram of Photoshot. Data were shown as the mean ± SEM (n = 5 rats/group). Con: control; DM: diabetes; Met: diabetes + 200 mg/kg per day metformin; Nano: diabetes + 465 mg/kg per day chitosan and silica; Nano-EE1: diabetes + 93 mg/kg per day EE; Nano-EE3: diabetes + 279 mg/kg per day EE; Nano-EE5: diabetes + 465 mg/kg per day EE. The values with different letters (a-d) are significantly different (*p* < 0.05) as analyzed by Duncan's multiple range test.

**Figure 17:**
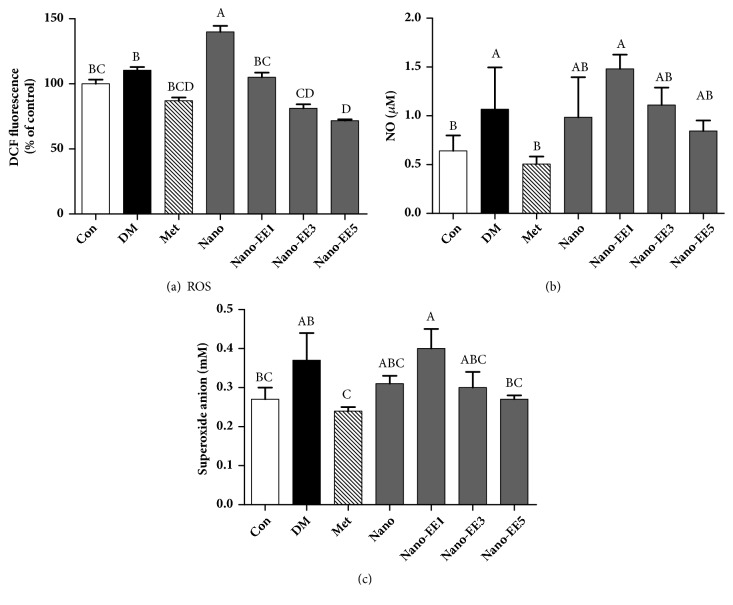
*Effects of nano-EE on the sperm production of ROS (a) and NO (b) superoxide anion (c) after 7 weeks of treatment in diabetic rats.* Data were shown as the mean ± SEM (n = 5 rats/group). Con: control; DM: diabetes; Met: diabetes + 200 mg/kg per day metformin; Nano: diabetes + 465 mg/kg per day chitosan and silica; Nano-EE1: diabetes + 93 mg/kg per day EE; Nano-EE3: diabetes + 279 mg/kg per day EE; Nano-EE5: diabetes + 465 mg/kg per day EE. The values with different letters (A-B) are significantly different (*p* < 0.05) as analyzed by Duncan's multiple range test.

Histological assessment of the testicular tissue showed atrophy of seminiferous tubule, loss of testicular cells (such as Leydig and Sertoli cells), sloughing of centrally located spermatozoa, and disappearance of spermatids in the seminiferous tubular lumen in both DM and nano groups (Figure 12). However, dose-dependent treatments of these animals with nano-EE for 7 weeks attenuated these changes and provided optimal protection at a dose of nano-EE5 (Figure 12). So, it is suggesting the protective action of nano-EE5 against oxidative stress-mediated testicular damage in diabetic rats.

The diameters of seminiferous tubules (DST), germinal cell layer thickness (GCLT), and the area of seminiferous tubules (AST) were significantly reduced, and the area of seminiferous lumen (ASL) was increased in DM group as compared to the Con, nano-EE3, and nano-EE5 (Table 5).

#### 3.4.4. Analysis of Anti-Inflammation

(*1) Effect of the Plasma TNF-α, IL-1β Inflammation Markers after 7 Weeks Treated with Nano-EE in Diabetic Rats.* A number of experimental and clinical data have clearly established that adipose tissue, liver, muscle, and pancreas are the sites of inflammation in the presence of type 2 diabetes mellitus [43]. Infiltration of macrophages into these tissues was seen in animal models of diabetes as well as in obese human individuals with metabolic syndrome or type 2 diabetes mellitus [43]. These cells are crucial for the production of proinflammatory cytokines, including TNF-*α*, IL-6, and IL-1*β*. They act in an autocrine and paracrine manner to promote the insulin resistance by interfering with insulin signaling in peripheral tissues through the activation of the c-JUN N-terminal kinase (JNK) and nuclear factor-kappa B (NF-*κ*B) pathways [43]. These pathways are activated in multiple tissues in type 2 diabetes mellitus and have a central role in promoting tissue inflammation. In the pancreas, activation of NLRP3 (NACHT, LRR, and PYD domains-containing protein 3) inflammasome by high levels of glucose and fatty acids and subsequent release of IL-1*β* lead to *β* cells dysfunction, apoptosis, insulin deficiency, and progression to type 2 diabetes mellitus [43]. In this study, the levels of IL-1*β* and TNF-*α* were increased in DM group but the levels were decreased in the Met and nano-EE5 groups.

**Figure 18:**
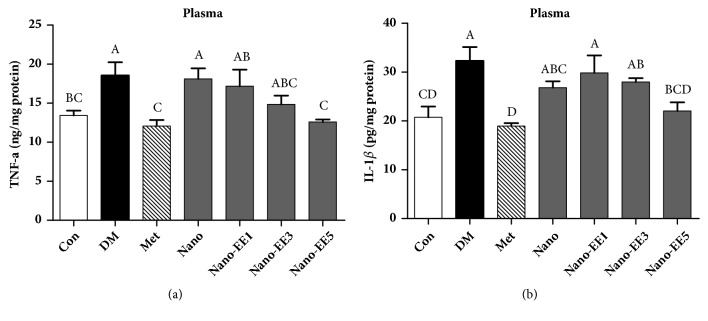
*Effect of the plasma TNF-α and IL-1β inflammation markers after 7 weeks of nano-EE treatment in diabetic rats.* Data were shown as the mean ± SEM (n = 5 rats/group). Con: control; DM: diabetes; Met: diabetes + 200 mg/kg per day metformin; Nano: diabetes + 465 mg/kg per day chitosan and silica; Nano-EE1: diabetes + 93 mg/kg per day EE; Nano-EE3: diabetes + 279 mg/kg per day EE; Nano-EE5: diabetes + 465 mg/kg per day EE. The values with different letters (A-B) are significantly different (*p* < 0.05) as analyzed by Duncan's multiple range test.

Results showed that, as compared to the Con group, TNF-*α* and IL-1*β* levels in the DM group were increased. A significant reduction in the level of TNF-*α* and IL-1 *β* was observed in Met and nano-EE5 groups and reached almost similar values to the Con group while no significant difference was observed between the Con, Met, and nano-EE5 groups (Figure 18).

## 4. Conclusion

In the present work, the observations suggested that the micro-nanoparticles prepared by an easy process without using high temperature and harsh organic chemicals might be employed to improve the potential oral delivery of EE. EE loaded chitosan/silica core-shell micro-nanoparticles (nano-EE) were characterized by DLS and SEM analysis and evaluated their effect both* in vitro* and* in vivo* system. The results indicated that the size of nano-EE was 218 ± 42 nm with an almost 66.9% encapsulation efficiency and 39.9% loading capacity. This chitosan/silica micro-nanoparticles enabled the oral delivery of EE with low toxicity.

Our* in vivo* study revealed that nano-EE treatment might provide effective protection against the oxidative damage in plasma and testis of STZ-induced type 2 diabetic rats. Since this compound was able to ameliorate the enzymatic and nonenzymatic antioxidant defense system to prevent the lipid peroxidation in the tissues, nano-EE5 improved oxidative stress, insulin resistance and restored liver and kidney function and mitochondrial membrane potential in diabetic rats.

Therefore, the micro-nanoencapsulated EE can be used as a promising tool for improving the reproductive function in diabetic rats and provide a smart way for the development of healthy products applicable to the pharmaceutical field.

## Figures and Tables

**Table 1:** The contents of the extraction yield, alkylamide, total phenolic acids, encapsulation rate, and loading capacity in freeze-dried EE and nano-EE were examined by HPLC analysis.

Quality Characteristics	Content	
	Nano-EE	EE
(1) Extraction yield (%)	-	7
(2) Total phenolic acids (mg/g freeze dried extract)	35.3	20.8
(3) Isobutylamides (mg/g freeze dried extract)	0.21	0.88
(4) Encapsulation efficiency (%)	66.9	-
(5) Loading capacity (%)	39.9	-

–: not detected.

**Table 2:** Particle size (PS), polydispersity index (PDI), and zeta potential (ZP) of formulated nano-EE by DLS.

	PS (nm)	PDI	ZP (mV)
Nano-EE	278 ± 21	0.37 ± 0.01	-21.3 ± 0.55

**Table 3:** Plasma insulin level, homeostasis model assessment equation (HOMA-IR), advanced glycation end products (AGEs), and plasma FGF 21 in diabetic rats fed different doses of nano-EE after 7 weeks.

	Con	DM	Met	Nano	Nano-EE1	Nano-EE3	Nano-EE5
Insulin (*μ*U/mL)	6.51 ± 0.05^c^	10.95 ± 0.03^ab^	7.91 ± 0.06^bc^	9.60 ± 0.07^abc^	11.65 ± 0.06^a^	9.95 ± 0.04^ab^	8.11 ± 0.02^bc^
HOMA-IR	1.10 ± 0.16^d^	3.47 ± 0.19^a^	2.06 ± 0.30^bc^	2.83 ± 0.44^ab^	3.48 ± 0.38^a^	2.69 ± 0.24^abc^	1.91 ± 0.08^c^
AGEs (*μ*g/ml)	86.9 ± 3.6^c^	115.4 ± 3.97^a^	85.4 ± 10.84^c^	126.3 ± 3.43^a^	120 ± 12.02^ab^	110.1 ± 2.78^abc^	93.9 ± 8.51^bc^
Plasma FGF 21 (ng/mg protein)	0.03 ± 0.01^b^	0.26 ± 0.01^a^	0.08 ± 0.01^b^	0.22 ± 0.01^ab^	0.21 ± 0.04^ab^	0.15 ± 0.02^ab^	0.05 ± 0.01^b^

Data were shown as the mean ± SEM (n = 5 rats/group). Con: control; DM: diabetes; Met: diabetes + 200 mg/kg per day metformin; Nano: diabetes + 465 mg/kg per day chitosan and silica; Nano-EE1: diabetes + 93 mg/kg per day EE; Nano-EE3: diabetes + 279 mg/kg per day EE; Nano-EE5: diabetes + 465 mg/kg per day EE. The values with different letters (a-b) represent significantly different (*p *< 0.05) as analyzed by Duncan's multiple range test.

HOMA-IR = fasting plasma glucose (mg/dL) × fasting plasma insulin (*μ*U/mL) / 405.

**Table 4:** Effects of plasma ALT, AST, BUN, and creatinine in diabetic rats fed different doses of nano-EE after 7 weeks.

	Con	DM	Met	Nano	Nano-EE1	Nano-EE3	Nano-EE5
AST (U/L)	12.47 ± 0.78^d^	20.04 ± 0.90^a^	15.78 ± 1.02^bc^	18.19 ± 1.14^ab^	13.13 ± 0.91^cd^	13.13 ± 0.74^cd^	12.33 ± 0.50^d^
ALT (U/L)	7.72 ± 0.69^b^	14.07 ± 0.39^a^	10.06 ± 0.58^b^	11.17 ± 1.55^ab^	11.98 ± 1.35^b^	10.20 ± 0.83^b^	9.01 ± 0.90^b^
BUN (mg/dL)	23.58 ± 1.77^b^	32.22 ± 8.46^ab^	27.35 ± 2.38^ab^	37.88 ± 5.60^a^	27.51 ± 2.28^ab^	24.20 ± 3.58^b^	23.58 ± 0.61^b^
Creatinine (mg/dL)	2.42 ± 0.06^bc^	2.92 ± 0.20^a^	2.66 ± 0.12^abc^	2.73 ± 0.16^ab^	2.88 ± 0.11^ab^	2.18 ± 0.16^cd^	1.93 ± 0.16^d^

Data were shown as the mean ± SEM (n = 5 rats/group). Con: control; DM: diabetes; Met: diabetes + 200 mg/kg per day metformin; Nano: diabetes + 465 mg/kg per day per chitosan and silica; Nano-EE1: diabetes + 93 mg/kg per day EE; Nano-EE3: diabetes + 279 mg/kg per day EE; Nano-EE5: diabetes + 465 mg/kg per day EE. The values with different letters (a-b) represent significantly different (*p *< 0.05) as analyzed by Duncan's multiple range test.

(i) Alanine aminotransferase (ALT).

(ii) Aspartate aminotransferase (AST).

**Table 5:** Effects of nano-EE and EE on diameters seminiferous tubules (DST), germinal cell layer thickness (GCLT), area of seminiferous tubules (AST), and area of seminiferous lumen (ASL) after 7 weeks of treatment.

	Con	DM	Met	Nano	Nano-EE1	Nano-EE3	Nano-EE5
(diameter Size)							
DST (*μ*m)	300.6 ± 15.1^a^	255.7 ± 5.9^c^	296.8 ± 7.7^a^	250 ± 4.4^c^	268 ± 3.6^bc^	288.7 ± 5.7^ab^	291.4 ± 4.0^a^
GCLT (*μ*m)	62.1 ± 0.8^a^	45.4 ± 0.9^c^	59.7 ± 1.2^a^	44 ± 1.0^c^	45.3 ± 1.5^c^	56.8 ± 0.9^b^	60.7 ± 2.0^a^

(Area)							
AST (%)	94.4 ± 1.0^a^	74.2 ± 2.2^bc^	93.5 ± 1.1^a^	71.7 ± 1.7^bc^	68.9 ± 4.6^c^	78.9 ± 4.6^b^	90.2 ± 1.7^a^
ASL (%)	5.6 ± 1.0^c^	25.8 ± 2.2^ab^	6.5 ± 1.1^c^	28.3 ± 1.7^a^	31.1 ± 4.6^a^	21.1 ± 4.6^b^	9.8 ± 1.7^c^

Data were shown as the mean ± SEM (n = 5 rats/group). Con: control; DM: diabetes; Met: diabetes + 200 mg/kg per day metformin; Nano: diabetes + 465 mg/kg per day per chitosan and silica; Nano-EE1: diabetes + 93 mg/kg per day EE; Nano-EE3: diabetes + 279 mg/kg per day EE; Nano-EE5: diabetes + 465 mg/kg per day EE. The values with different letters (a-c) represent significantly different (*p *< 0.05) as analyzed by Duncan's multiple range test. The area % was calculated by the formula, where As is area covered by seminiferous tubules or lumen. T is total area of the field. % As = As × 100.

## Data Availability

No data were used to support this study. The statements that supporting our findings are cited in references.
